# Development and validation of a dual-purpose 60K single nucleotide polymorphism chip for simultaneous genotyping of chicken and duck

**DOI:** 10.5713/ab.250809

**Published:** 2025-12-18

**Authors:** Jinhyeong Kim, Eunjin Cho, Minjun Kim, Jaewon Kim, Dongwon Seo, Jung-Woo Choi, Won-Hyong Chung, Yeongkuk Kim, Hyojun Choo, Jun Heon Lee

**Affiliations:** 1 Department of Animal Science, Chungnam National University, Daejeon, Korea; 2 Department of Bio-AI Convergence, Chungnam National University, Daejeon, Korea; 3 Poultry Research Center, National Institute of Animal Science, Rural Development Administration, Pyeongchang, Korea; 4 Research Institute TNT Research Company, Jeonju, Korea; 5 Department of Animal Science, College of Animal Life Sciences, Kangwon National University, Chuncheon, Korea; 6 Quantomic Research & Solution Co., TIPS Town, Daejeon, Korea

**Keywords:** Chicken, DNA Pooling, Duck, Genotyping, Single Nucleotide Polymorphism, Single Nucleotide Polymorphism Chip

## Abstract

**Objective:**

Chickens and ducks represent the most important poultry species within the livestock industry. However, the availability of single nucleotide polymorphism (SNP) chips for these species is limited, and development of separate chips for each species requires considerable expense. To address this limitation, we developed the first poultry SNP chip applicable to both species simultaneously. The 60K SNP chip comprises 30,816 SNPs for chickens and 35,209 SNPs for ducks. The performance of the chip was evaluated using non-pooled datasets (chicken only and duck only) and a pooled dataset combining chicken and duck DNA to verify that genotyping accuracy was maintained without cross-species interference.

**Methods:**

DNA was extracted from 28 Korean native chickens and 28 Korean native ducks. Genotyping was performed using the Illumina platform, employing chicken-only, duck-only, and pooled chicken-duck SNP panels. Genotype accuracy and concordance were evaluated with PLINK. Sample and SNP quality were assessed in accordance with the Illumina genotyping protocol. Principal component analysis (PCA) was conducted to evaluate and compare the chicken-duck pooled dataset with the non-pooled datasets.

**Results:**

The mean genotype concordance of non-pooled (chicken only and duck only) datasets exceeded 99%, while concordance between non-pooled and chicken-duck pooled datasets surpassed 97.8%. All datasets satisfied quality thresholds for call rate, GenCall score, 10% GenCall score, and cluster separation. PCA revealed consistent clustering patterns, with no significant differences observed between non-pooled and pooled datasets.

**Conclusion:**

The first, at the best of our knowledge, poultry SNP chip incorporating chicken-duck pooled samples, has been successfully developed and validated. This chip provides reliable genotyping performance for both species and presents a cost-effective option for large-scale SNP chip development in the livestock industry.

## INTRODUCTION

The development of single nucleotide polymorphism (SNP) chips has provided an inexpensive and reliable tool for genotyping thousands of samples, which is crucial for breeding programs as well as the management of wild and indigenous populations. SNP chips are widely used in genome-wide association studies (GWAS) to identify candidate genes associated with diverse traits. Nevertheless, one limitation of SNP chips is that the identification of variants and the design of efficient SNP chips depend on the availability of highly accurate and well-annotated reference genomes [1,2]. To overcome these limitations, high-density genotyping technologies such as whole-genome sequencing (WGS) and third-generation sequencing have been increasingly adopted [3–5]. However, these approaches remain costly when screening large numbers of samples, particularly in species with large genome sizes. Therefore, SNP chips are still widely used alongside WGS across animal industries [6,7]. Numerous single-species SNP chips have been developed to analyze the population structure, detect trait-associated variants, and enable genomic selection in livestock [6,8,9]. Moreover, various SNP chips with different marker densities have been designed for plants and animals, including wild species. Nevertheless, single-species chips remain relatively expensive.

To address these limitations, previous studies have explored custom SNP chips combining related or closely associated species or populations, such as the Illumina 9K SNP chip for pear (*Pyrus communis*) and apple (*Malus domestica*) [10], the Axiom 60K SNP chip for Pacific (*Crassostrea gigas*) and European oysters (*Ostrea edulis*) [11], and the Axiom 60K SNP chip developed for four species (*Rubus*, *Mānuka*, *Snapper*, *Trevally*) using pooled genotypes to validate quality [12]. Despite these efforts, SNP chips incorporating pooled DNA for livestock species remain scarce.

Chicken (*Gallus gallus*) is one of the most economically important livestock species worldwide. Chicken meat is more economical than other meat sources due to its short production cycle and relatively low cost. In 2019, a total of meat production reached 207 million tons, of which chicken accounted for 57% [13]. In the same year, laying hens produced approximately 1,577 billion eggs, emphasizing their economic significance. SNP chips of varying densities have been developed and extensively applied to facilitate genetic studies in chickens. For example, a 50K SNP chip was specifically designed for egg-type chickens [14,15], whereas a high-density 600K SNP chip has been used, through imputation, to investigate carcass traits using GWAS [16]. Overall, SNP chips ranging from medium to high density have been widely utilized for GWAS and other genomic analyses [16–18].

Ducks (*Anas platyrhynchos*) are another economically important poultry species worldwide, particularly in Asia and other developing regions. Ducks play a crucial role in meeting daily protein requirements, with Asian countries accounting for 84.2% of global duck production [19]. However, unlike chickens, the availability of duck SNP chips remains limited. The classification of ducks are a non-model species, coupled with their limited genomic resources, faces inherent challenges in the development of SNP chips. The current duck reference genome is notably less complete and less accurately annotated compared with those of model species, resulting in fewer validated genetic variants available for chip design. Therefore, the development of additional SNP chips for ducks is crucial.

In this circumstance, multispecies SNP chips offer a solution by substantially reducing the time and cost required to design and manufacture separate single-species chips. Furthermore, the use of pooled DNA provides an effective strategy for analyzing large numbers of samples in a cost-efficient manner. Given the increasing demand for rapid and accurate analysis of large populations in the breeding industry, particularly for non-model species, the development of efficient genotyping tools is urgent. Therefore, the development of a dual-species SNP chip represents an essential strategy, as it can reduce costs and time substantially, while enabling the analysis of large numbers of samples in livestock research and breeding programs.

In this study, we developed the first poultry 60K SNP chip containing chicken and duck SNPs obtained from pooled samples. The developed chip was validated by comparing genotypes across SNP types (pooled and non-pooled) and assessing their quality according to the Illumina protocol. Furthermore, we evaluated the performance of non-pooled and pooled samples using principal component analysis (PCA) to assess its applicability. This study provides a valuable genomic tool for future research in poultry breeding and the livestock industry.

## MATERIALS AND METHODS

**Figure 1:**
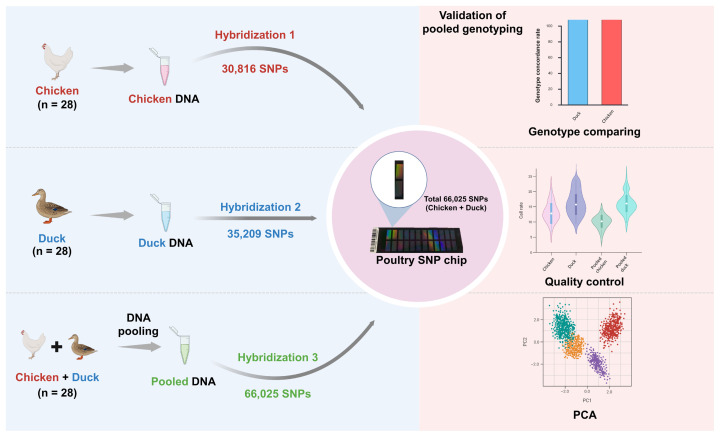
An overview of the study validation workflow for the 60K poultry SNP chip. The process involved the three hybridization experiments using different sample sets: DNAs from chicken only samples (Hybridization 1), DNAs from duck only samples (Hybridization 2), and DNAs from chicken-duck pooled samples (Hybridization 3). The performance of the chip was validated through genotype comparison, quality control assessment, and principal component analysis to confirm its applicability, particularly for the chicken-duck pooled genotype dataset. SNP, single nucleotide polymorphism; PCA, principal component analysis.

The overall workflow of this study is summarized in Figure 1. The workflow includes DNA extraction, DNA pooling, SNP genotyping, genotype comparison, quality control (QC), and PCA.

### DNA extraction & DNA pooling

Genomic DNA was extracted from blood samples of Red-brown Korean native chickens and Korean native ducks using PrimePrep DNA Extraction Kit (GenetBio). For pooled samples, DNA from chickens and ducks was mixed at a 1:1 ratio for each set of 28 individuals. Prior to pooling, DNA concentration and integrity were measured to ensure that low-quality or low-concentration samples did not contribute to potential cross-species interference during genotyping.

### Single nucleotide polymorphism selection

The SNPs for Korean native ducks were selected using the criteria of a genotype call rate>0.9, minor allele frequency (MAF)>0.2, and linkage disequilibrium (LD) with R^2^<0.4. A total of 36,609 SNP markers were retained, which included 591 SNPs from 33 duck quantitative trait loci (QTL)-associated genes. Among these 36,609 SNPs, genotypes were successfully identified for 35,209 SNPs.

For Korean native chickens, 30,816 SNPs were selected based on an imputation accuracy>0.9 using this custom 60K SNP chip [20]. A custom 60K SNP chip for indigenous chickens was developed consisting of 61,242 SNPs. This chip included 12,289 imputation-bridge SNPs compatible with the Illumina 60K SNP chip and 37,076 imputation-bridge SNPs compatible with the Affymetrix 600K SNP chip. In detail, the custom chip contained 11,880 SNPs located in trait associated genomic regions, 150 markers for distinguishing indigenous breeds, and 342 markers targeting the MHC region.

### Single nucleotide polymorphism chip genotyping

Genotyping was conducted using two methods based on the Illumina poultry 60K SNP chip (Figure 1). The first method analyzed non-pooled samples of chickens and ducks separately, while the second method involved chicken-duck pooled DNA to evaluate the applicability of pooled genotype data.

For the chicken panel, 28 chicken samples and 30,816 SNPs from a custom 60K SNP chip [20] were used after QC. For the duck panel, 35,209 SNPs were used from the duck whole-genome resequencing data [21]. However, due to insufficient DNA in some samples, different individuals from the resequencing dataset were used. We selected 28 samples that passed QC and were collected in ascending order of sample numbers. The same SNPs and samples used in the non-pooled (chicken only and duck only) datasets were also subjected to the chicken-duck pooled SNP dataset. In total, 28 chicken samples with 30,816 SNPs and 28 duck samples with 35,209 SNPs were included for pooled and non-pooled genotyping datasets (Figure 1).

All samples and SNPs were randomly genotyped on a SNP chip without dividing the chip into separate regions for each sample type.

### Validating single nucleotide polymorphism genotype concordance

To evaluate the performance of the 60K poultry SNP chip, genotype concordance was assessed for each SNP dataset (Figure 1). For non-pooled (chicken only) SNPs and the custom 60K chip, concordance was calculated using PLINK 1.9 with the --bmerge and --merge mode 7 options [22]. Since this option can only be applied to identical samples and SNP sets, it was not applicable for non-pooled (duck only) SNPs and whole-genome resequencing data. Common SNPs between whole-genome resequencing and non-pooled (duck only) dataset aligned as PLINK B-files. In these cases, genotypes and allele concordance were determined based on chromosome position and SNP identity.

After the evaluation of genotype concordance for non-pooled (chicken only and duck only) SNPs, the chicken-duck pooled SNP dataset was analyzed using the same PLINK 1.9 --bmerge and --merge mode 7 options. Genotyping accuracy was estimated based on the formula shown below:


(1) $$Concordance\;rate\;(\%)=\frac{N_{total}-N_{diff}}{N_{total}}\times 100,$$

*N_total_* represents the total number of alleles on each SNP dataset, and *N_diff_* represents the number of mismatched alleles identified during the merge process.

### Validating the quality of samples

To validate the quality of genotyped samples, we applied the Illumina sample QC protocol. The first method was based on the call rate score. This score ranges from 0 to 1, representing the proportion of successfully called genotypes for each sample. We applied a threshold of 0.95 according to the Infinium genotyping data analysis guide [23,24]. The next method involves the 10% GenCall (GC) score, which is used to identify low-quality samples. Samples with a 10% GC score below the threshold were either reprocessed or discarded. We applied a threshold of 0.3, following the genotyping data analysis guide [23].

### Validating the quality of single nucleotide polymorphisms

We applied the Illumina SNP QC procedure to validate the quality of genotyped SNPs. The first method was assessed based on GC score cutoff. The GC, which ranges from 0 to 1, is calculated for each genotype and indicates the distance of a sample from the center of its cluster. Lower values represent genotypes located further from the cluster center. Scores below 0.2 indicate failed genotypes, while scores above 0.7 are generally considered well-genotyped SNPs [25]. According to the Illumina FastTrack Genotyping Service guidelines, a threshold of 0.15 is typically used [23]. Genotypes with a GC score below 0.15 were considered unreliable and excluded from further analysis. The next method was based on the Cluster Separation (Cluster Sep) score. Cluster Sep measures the degree of separation between three genotypes (0/0, 0/1, 1/1) clusters and is used to evaluate whether clusters overlap for individual SNPs. The score ranges from 0 to 1. According to the Infinium genotyping data analysis guide, we applied a threshold of 0.3 for Cluster Sep score [23,24].

### Quality control of genotyping data

SNP genotype data of the developed 60K poultry chip were called using GenomeStudio v2.0 Software (Illumina) with a standard QC procedure [24]. The QC process was divided into two levels: sample-level and SNP-level. In the first step, poorly genotyped samples were removed if they had a call rate<95% or a p10 GC<0.3. Using the retained samples, the second step involved filtering out low-quality SNPs with a GC score<0.15 or a Cluster Sep score<0.3. After the GenomeStudio QC process, the remaining samples and SNPs were retained for subsequent analyses. For further QC, an additional filtering step was conducted using PLINK 1.9. SNPs were excluded if they had a genotype call rate<0.9, a MAF<0.01, and a Hardy-Weinberg equilibrium (HWE) p-value<1×10^−6^.

### Principal component analysis of genotyping data

PCA was conducted using the genotypes and samples retained after the QC steps to assess the genetic distance among groups and to examine potential differences between pooled chicken-duck and non-pooled data.

## RESULTS AND DISCUSSION

### Genotyping accuracy of single nucleotide polymorphism chips

We evaluated the genotype accuracy of the developed 60K poultry SNP chip, which produced three datasets from the same chip: the non-pooled panels (chicken only and duck only) and the chicken-duck pooled panel.

For chickens, the accuracy of the chicken genotypes from chicken only dataset was assessed by comparison with the custom 60K SNP chip data. 28 samples and 30,816 SNPs common to both datasets were selected for analysis. Genotype concordance was evaluated using PLINK 1.9 with the --bmerge and --merge-mode 7 options. Among the 30,816 SNPs, 30,787 were concordant and 29 were discordant, corresponding to an overall concordance rate of 99.91% (Table 1). Specifically, 847,250 out of 862,036 alleles were identical between the two datasets. These results demonstrate that the non-pooled chicken only dataset provides reliable genotyping data. To further validate pooled genotypes, we compared the chicken genotypes from chicken only dataset with the chicken genotypes extracted from the chicken-duck pooled dataset. Using the same 28 samples with 30,816 SNPs, 30,369 SNPs were concordant and 223 SNPs were discordant, corresponding to a concordance rate of 99.27% (Table 1). At the allele level, 856,759 out of 862,848 alleles were concordant. Thus, non-pooled and pooled chicken datasets exhibited concordance rates exceeding 99%, confirming that the pooled genotyping data are sufficiently reliable for downstream analyses.

For ducks, the accuracy of duck genotypes from non-pooled duck only was evaluated by comparison with duck whole-genome resequencing data. A total of 35,209 SNPs were extracted based on genomic positions. Of these, 34,549 SNPs were concordant, and 712 SNPs were discordant, corresponding to a concordance rate of 98.13% (Table 1). These results indicate that the non-pooled duck only SNP dataset was accurately genotyped. However, we compared genotype based on We then compared the duck genotypes from duck only dataset with duck genotypes from the chicken-duck pooled dataset. Using PLINK v1.9, we analyzed 28 samples with 35,209 SNPs, of which 34,434 were concordant and 1,276 were discordant. At the allele level, 916,600 out of 950,124 alleles were concordant, corresponding to an overall concordance rate of 97.8%. These results demonstrate that both non-pooled and pooled duck datasets achieved high genotyping accuracy.

For the discordant SNPs, we examined the BSC files for chickens (chicken only and chicken-duck pooled) and duck (duck only and chicken-duck pooled) using Genome studio 2.0. Based on GenTrain algorithm, genotype calls are determined based on two dye intensity signals (A and B) [26]. However, when the signals are low or ambiguous, the GenTrain algorithm is unable to clearly separate clusters or assign genotypes accurately. In our analyses, several samples corresponding to the mismatched SNPs identified in the diff file showed ambiguous patterns. These discrepancies are likely attributable to low DNA concentration or interference between DNA samples during hybridization. Therefore, further investigation is required to clarify the causes of discordant SNPs. However, the proportion of discordant SNPs in our study was very low, indicating that these issues had only a minimal impact on overall genotyping quality.

In conclusion, all four datasets (chicken genotypes from chicken only, duck genotypes from duck only, chicken genotypes from chicken-duck pooled, duck genotypes from chicken-duck pooled) exhibited high concordance rates, confirming that the SNP chip provides reliable genotyping data suitable for downstream analyses.

### Sample quality assessment

**Figure 2:**
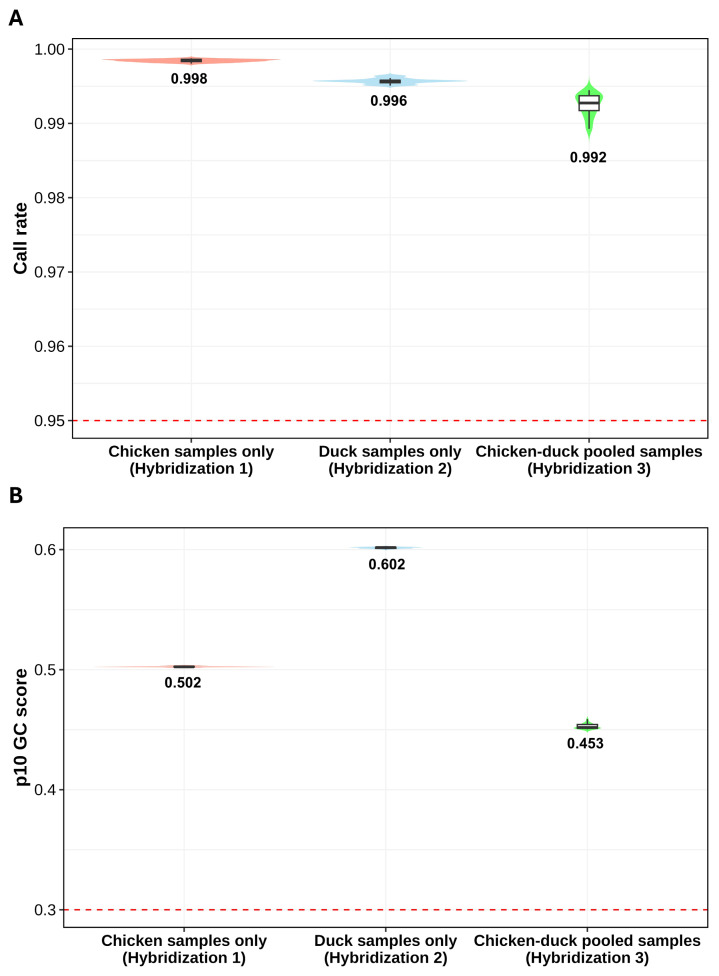
Comparison of genotyping quality scores between chicken only (Hybridization 1), duck only (Hybridization 2), and chicken-duck pooled (Hybridization 3) samples. (A) Violin and box plots for call rates. (B) Violin and box plots for 10% GenCall (p10 GC) scores. The dashed red line in each panel indicates the minimum quality threshold (0.95 for call rate and 0.3 for p10 GC score). The mean score for each sample group is displayed on the plots. All sample groups successfully exceeded the quality control thresholds for both scores, which indicates the good quality of the genotyped samples.

The quality of genotyped samples was assessed for both non-pooled and pooled datasets following the Illumina genotyping data analysis protocol [23]. All samples exceeded the recommended threshold of 0.95 (Figure 2A). Specifically, chicken samples only exhibited a mean call rate of 0.998 (standard deviation [SD] = 0.00019), duck samples only had a mean call rate of 0.996 (SD = 0.00036), and chicken-duck pooled samples showed a mean call rate of 0.992 (SD = 0.0015). These results indicate that all samples (chicken samples only, duck samples only, and chicken-duck pooled samples) were successfully genotyped with high accuracy.

The second QC method involved assessment of the 10% GC score, which is used to identify low-quality samples recommended for re-clustering. All samples exceeded the threshold (Figure 2B), with mean 10% GC scores of 0.502 (SD = 0.0005) for chicken samples only, 0.602 (SD = 0.0006) for duck samples only, and 0.453 (SD = 0.0002) for chicken-duck pooled samples. As all values were above the 0.3 threshold, both the non-pooled and pooled datasets were reliably genotyped, with no significant differences were observed among the sample types.

In conclusion, all three sample types (chicken samples only, duck samples only, and chicken-duck pooled samples) were successfully genotyped and well-clustered.

However, during the QC process, pooled samples inherently contain relatively higher risk of variability compared with non-pooled types. Variations in DNA concentration, degradation levels, or unequal representation of individuals within a pool may affect hybridization efficiency and cluster separation. Therefore, careful standardization of DNA quality and ensuring equal DNA contribution from all individuals are essential to minimize variability when applying DNA pooled genotyping.

### Single nucleotide polymorphism quality assessment

**Figure 3:**
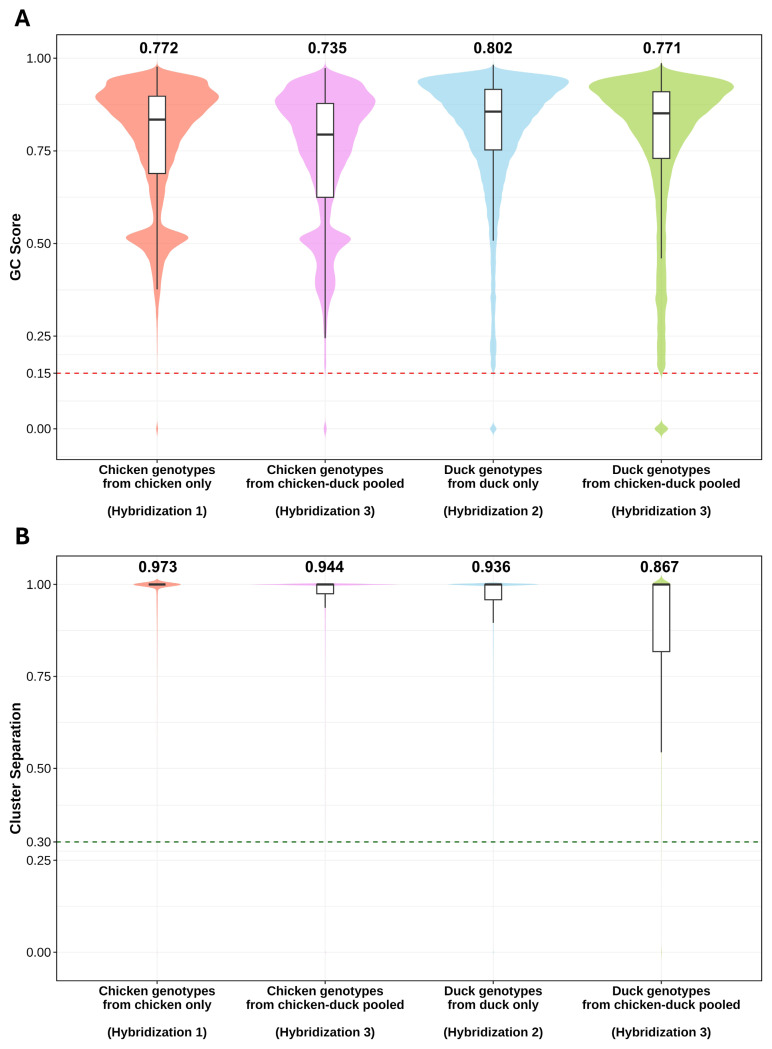
Comparison of genotyping quality scores between chicken genotypes from chicken only (Hybridization 1) dataset, chicken genotypes from chicken-duck pooled (Hybridization 3) dataset, duck genotypes from duck only (Hybridization 2) dataset, and duck genotypes from chicken-duck pooled (Hybridization 3) dataset. (A) Violin and box plots of GenCall (GC) scores. (B) Violin and box plots of cluster separation scores. The dashed red lines represent the minimum quality threshold (0.15 for GC and 0.3 for cluster separation score). The mean score for each genotype group is displayed on the plots. All genotype groups successfully exceeded the quality control thresholds for both scores, which indicates the good quality of the genotyped SNPs. SNP, single nucleotide polymorphism.

The quality of genotyped SNPs was evaluated to assess their reliability. The first QC method was based on the GC score, and SNPs with a GC score below 0.15 were discarded. All SNPs exceeded this threshold (Figure 3A). Specifically, for chickens, the mean GC score of genotypes from non-pooled chicken only dataset was 0.772 (SD = 0.166), while chicken genotypes extracted from the chicken-duck pooled dataset had a mean GC score of 0.735 (SD = 0.183). Mean GC scores of the both groups were similar and exceeded 0.7, indicating that these genotypes were well-genotyped [7]. For ducks, the mean GC score of genotypes from non-pooled duck only dataset was 0.802 (SD = 0.169), and duck genotypes extracted from the chicken-duck pooled dataset had a mean score of 0.771 (SD = 0.207). These values also exceeded 0.7, confirming the high genotyping quality of both groups. Overall, non-pooled and pooled genotypes exhibited comparable genotyping quality.

The second QC method was based on Cluster Sep scores, with genotypes below a score of 0.3 recommended for re-clustering. All genotypes, both non-pooled and pooled, exceeded this threshold (Figure 3B). For chickens, the mean Cluster Sep score of genotypes from non-pooled chicken only dataset was 0.973 (SD = 0.085), whereas chicken genotypes extracted from chicken-duck pooled dataset had a mean score of 0.944 (SD = 0.138). No substantial difference was observed between the two groups. For ducks, the mean Cluster Sep score of genotypes from non-pooled duck only dataset was 0.936 (SD = 0.148), and duck genotypes extracted from chicken-duck pooled panel had a mean score of 0.867 (SD = 0.215). These results confirmed that both non-pooled and pooled SNPs were well clustered.

In conclusion, all four SNP groups (chicken genotypes from chicken only, chicken genotypes from chicken-duck pooled, duck genotypes from duck only, and duck genotypes from chicken-duck pooled) were well-genotyped and well-clustered, with no significant differences observed between non-pooled and pooled datasets.

### Quality control and additional analyses

**Figure 4:**
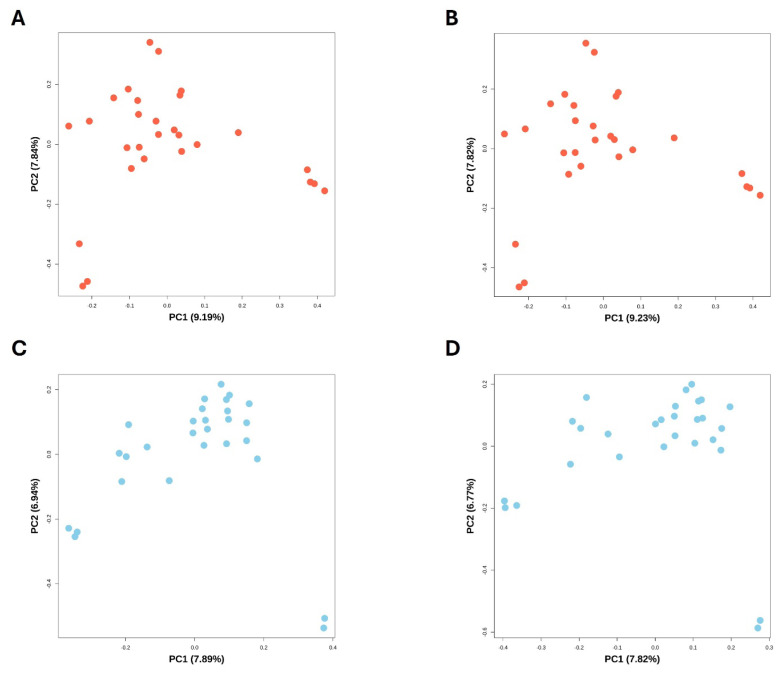
Principal component analysis (PCA) of chicken and duck populations. (A) PCA of the chicken only population. (B) PCA of chicken samples extracted from the chicken-duck pooled population. No significant differences between non-pooled (chicken only) and pooled populations. (C) PCA of the duck only population. (D) PCA of duck samples extracted from the chicken-duck pooled population. No significant differences between non-pooled (duck only) and pooled populations. The PC1 and PC2 axes represent percentages of explained variance. These results demonstrate that there were no significant differences between the non-pooled (chicken only and duck only) datasets and chicken-duck pooled datasets, and that the applicability of the pooled data for population analysis.

The QC procedure followed the Illumina genotyping protocol and PLINK, with detailed thresholds described in the Materials and Methods section. The PCA results visualized the genetic structure of chicken and duck populations (Figure 4). Each of the four PCA plots represents 28 samples from the respective chicken and duck populations, and each point in the plot corresponds to a single individual.

For non-pooled chicken only genotypes, 26 SNPs were discarded according to the Illumina QC protocol. No samples were excluded based on call rate or 10% GC score. However, 25 SNPs were removed due to Cluster Sep, and one SNP due to GC score. Following Illumina QC, 30,790 SNPs were retained for further analysis. Subsequent PLINK QC removed an additional 109 SNPs for low genotype call rate, 63 SNPs for deviation from HWE, and 4,813 SNPs for low MAF, leaving 25,805 high-quality SNPs for PCA (Figure 4A).

For the chicken-duck pooled genotypes, 713 SNPs were discarded from a total of 66,025 during Illumina QC. No samples were excluded based on call rate or 10% GC score; however, 693 SNPs were excluded due to low Cluster Sep, and 20 SNPs due to GC score. This resulted in 65,312 SNPs, which were then separated into pooled chicken and duck datasets based on genomic positions.

For the chicken genotypes from the chicken-duck pooled dataset, PLINK QC further identified and removed 166 SNPs with low genotype call rate, 121 SNPs for HWE deviation, and 4,806 SNPs with low MAF. Following the removal of redundant data, 25,659 SNPs remained for PCA (Figure 4B).

In Figure 4A, PC1 and PC2 explained 9.19% and 7.84% of the total genetic variance. Although the samples originated from a single population, the 28 individuals displayed separation, which can be attributed to individual genetic variations. In Figure 4B, PC1 and PC2 explained 9.23% and 7.82% of the total genetic variance. The clustering pattern observed in Figure 4B was consistent with that of Figure 4A. Comparison of PCA results between non-pooled and pooled chicken datasets revealed no substantial differences, indicating that both datasets are suitable for analyses in chicken populations.

For the non-pooled duck only dataset, no samples were excluded during the sample QC. However, 159 SNPs were removed due to inadequate Cluster Sep, and 12 SNPs were excluded due to the low 10% GC score, leaving 35,038 SNPs. PLINK QC then discarded 285 SNPs with low genotype call rate, 395 SNPs for HWE deviation, and 915 SNPs with low MAF, resulting in 33,443 high-quality SNPs for PCA (Figure 4C).

For duck genotypes from the chicken-duck pooled dataset, PLINK QC removed a total of 2,369 SNPs, including 754 with low genotype call rate, 495 with HWE deviation, and 1,120 with low MAF. PCA was performed on the remaining 32,214 SNPs (Figure 4D). A comparison of PCA results between non-pooled and pooled duck datasets revealed no substantial differences, demonstrating that the pooled dataset is also suitable for further analyses in duck populations.

In Figure 4C, PC1 and PC2 explained 7.89% and 6.94% of the total genetic variance. Consistent with the chicken results, duck samples despite originating from a single population, showed separation due to individual genetic diversity. In Figure 4D (PC1 = 7.82%, PC2 = 6.77), minor variations in PC values were observed due to differences in the remaining SNPs. However, the cluster pattern was conserved. Consequently, comparison of PCA results between non-pooled and pooled duck datasets revealed no substantial differences, indicating that both datasets are suitable for analyses in duck populations.

In conclusion, as shown in Figure 4, analyses using both non-pooled and pooled datasets produced concordant PCA results, confirming that pooled genotypes and samples are reliable and suitable for downstream analyses.

## CONCLUSION

We developed a 60K poultry SNP chip that integrates both chicken and duck samples. To evaluate the reliability of pooled genotypes, we compared them with their corresponding non-pooled datasets and confirmed that SNPs were accurately genotyped, ensuring suitability for downstream analyses. These findings demonstrate that the developed poultry SNP chip provides reliable genotyping performance across poultry populations. Furthermore, this development is expected to reduce the cost of large-scale genotyping in the livestock industry.

## Figures and Tables

**Table 1:** Concordance rates of SNP genotypes of non-pooled (chicken only and duck only) and chicken-duck pooled datasets

Comparison	Total SNPs	Matched SNPs	Mismatched SNPs	Concordance rate (%)
Chicken genotypes from chicken only vs. Custom 60K genotypes^1)^	30,816	30,787	29	99.91
Chicken genotypes from chicken-duck pooled dataset vs. Chicken genotypes from chicken only dataset^2)^	30,816	30,369	223	99.27
Duck genotypes from duck only dataset vs. WGS data^3)^	35,209	34,549	712	98.13
Duck genotypes from chicken-duck pooled dataset vs. Duck genotypes from duck only dataset^4)^	35,209	33,933	1276	97.80

1) Genotypes from chicken only dataset (Hybridization 1) were compared with custom 60K chip genotypes to assess genotype accuracy.

2) Chicken genotypes from the chicken-duck pooled dataset (Hybridization 3) were compared with those from the chicken only dataset to evaluate the accuracy and applicability of the pooled dataset.

3) Genotypes from the duck only dataset (Hybridization 2) were compared with WGS data to validate genotype accuracy.

4) Duck genotypes from the chicken-duck pooled dataset (Hybridization 3) were compared with those from the duck only dataset to evaluate the accuracy and applicability of the pooled dataset.

SNP, single nucleotide polymorphism; WGS, whole-genome resequencing.

## Data Availability

The data supporting the findings of this study are publicly available on Figshare at the following link: 10.6084/m9.figshare.30406429.
